# Supplementary material for: Feasibility and acceptability of collecting dried blood spots (DBS) from children after vaccination during supplementary immunization activities to estimate measles and rubella seroprevalence
Source: PLOS Glob Public Health. 2024 Jun 28;4(6):e0002985. doi: 10.1371/journal.pgph.0002985 (PMC11213301; doi:10.1371/journal.pgph.0002985)
Supplement: S1 Table — In recruitment of participants, we tried to ensure a mix of fixed and outreach vaccination sites were represented, no overlap in team members (both team members from the same study team not included) and included study team staff and vaccination campaign team staff working at the same health facility. (DOCX) [file pgph.0002985.s001.docx]

S1 Table. Characteristics of staff participating in qualitative interviews

|  | Choma (N=23) | Ndola  (N=19) | Total  (N=42) |
| --- | --- | --- | --- |
| Staff category |  |  |  |
| Serosurvey supervisor | 3 | 3 | 6 |
| Serosurvey data collector | 10 | 8 | 18 |
| Vaccinator | 10 | 8 | 18 |
| First involvement with a vaccination campaign^a^ | 4 | 8 | 12 |
| Usual employment^a,b^ |  |  |  |
| Formal health workers | 19 | 16 | 35 |
| Informal health workers | 1 | 0 | 1 |

1. Restricted to serosurvey data collectors and vaccinators.
2. Formal health workers include professional health and medical personnel such as nurses, midwives, and clinical officers. Informal health workers are community-based volunteers.

In recruitment of participants, we tried to ensure a mix of fixed and outreach vaccination sites were represented, no overlap in team members (both team members from the same study team not included) and included study team staff and vaccination campaign team staff working at the same health facility.
