# Supplementary figures and images for: Feasibility and acceptability of collecting dried blood spots (DBS) from children after vaccination during supplementary immunization activities to estimate measles and rubella seroprevalence
Source: PLOS Glob Public Health. 2024 Jun 28;4(6):e0002985. doi: 10.1371/journal.pgph.0002985 (PMC11213301; doi:10.1371/journal.pgph.0002985)

**S1 Figure. Number of children enrolled by campaign day and site**


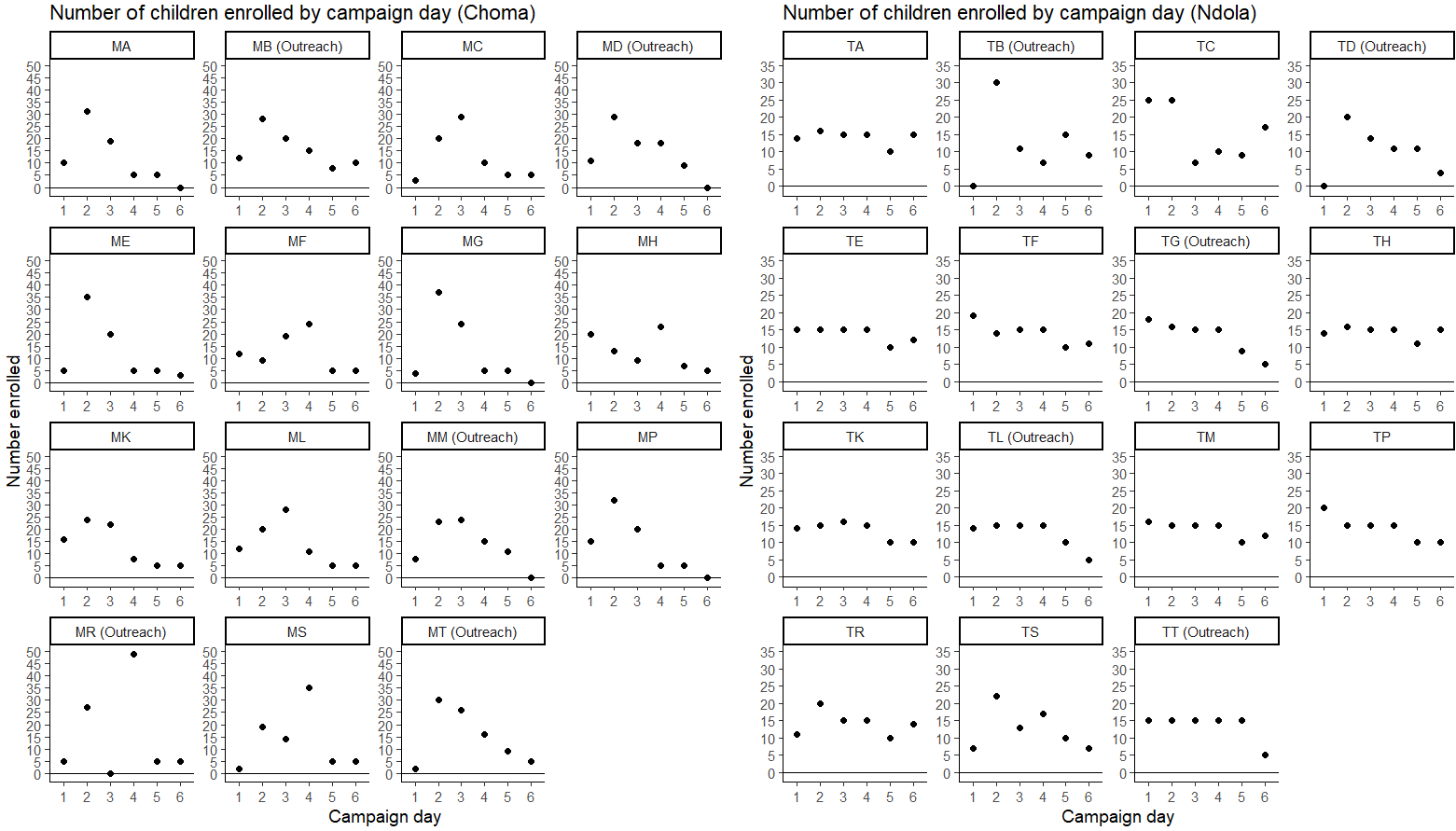

Supplement: S1 Fig — (DOCX) [file pgph.0002985.s002.docx]
