# Supplementary material for: Feasibility and acceptability of collecting dried blood spots (DBS) from children after vaccination during supplementary immunization activities to estimate measles and rubella seroprevalence
Source: PLOS Glob Public Health. 2024 Jun 28;4(6):e0002985. doi: 10.1371/journal.pgph.0002985 (PMC11213301; doi:10.1371/journal.pgph.0002985)
Supplement: S2 Fig — Data on the sampling interval was missing at some timepoints. (DOCX) [file pgph.0002985.s003.docx]

**S2 Figure. Sampling interval by campaign day and site**


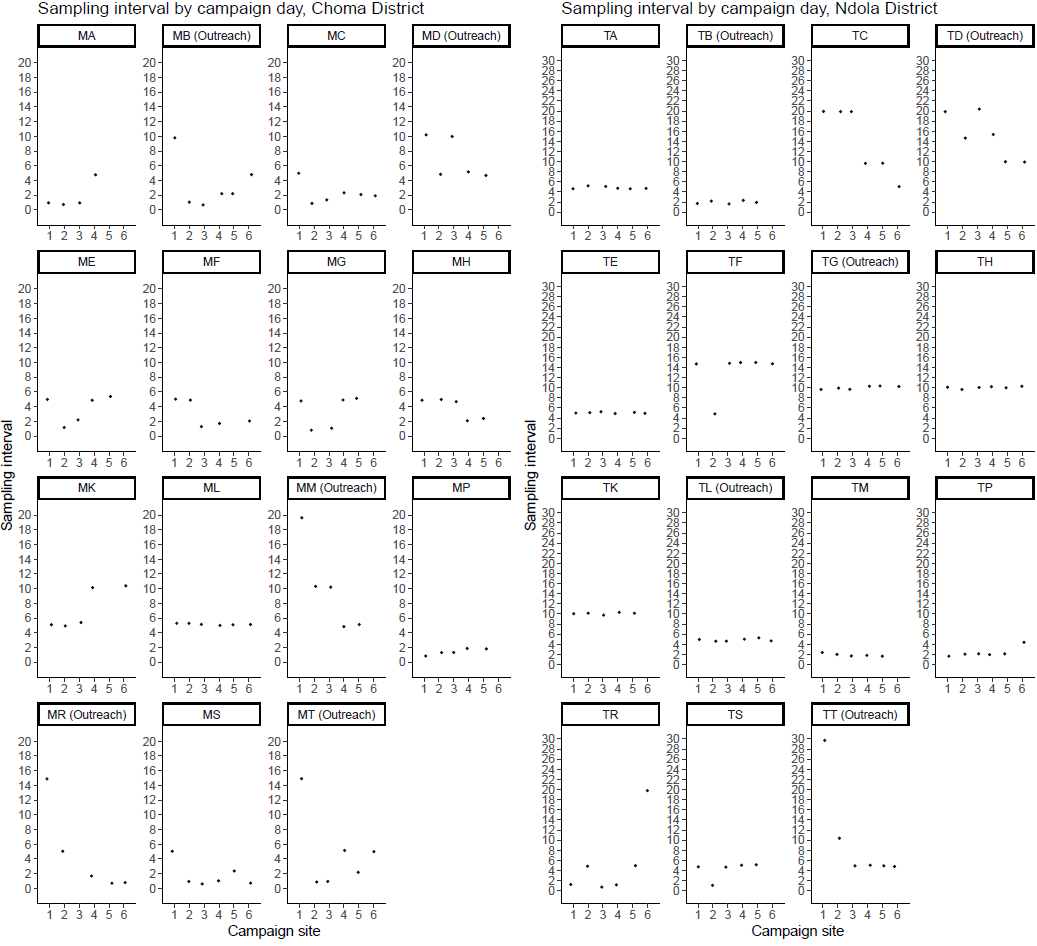


Footnote: Data on the sampling interval was missing at some timepoints.
