# Supplementary material for: Feasibility and acceptability of collecting dried blood spots (DBS) from children after vaccination during supplementary immunization activities to estimate measles and rubella seroprevalence
Source: PLOS Glob Public Health. 2024 Jun 28;4(6):e0002985. doi: 10.1371/journal.pgph.0002985 (PMC11213301; doi:10.1371/journal.pgph.0002985)

**S1 Appendix. Supplemental Methods**

Covid-19 safety precautions

The flow of participants at each vaccination site was controlled due to the COVID-19 pandemic. The vaccination team at each site ensured that there was social distancing and attendees were wearing masks. Masks were provided to those who did not have any. All staff also wore masks.

Sampling Log

Sampling logs with varying sampling intervals (e.g., every 5^th^, every 10^th^, every 20^th^) were developed as tools to aid survey staff in systematic sampling of children attending the SIA. The survey staff selected the log that corresponded to the sampling interval at their facility for that day. Each row in the log represented a sampling group or wave (e.g., child count 1-10 for every 10^th^ sampling interval). As children were vaccinated, the survey staff ticked off the boxes to indicate the child was counted but not selected. Once they reached the specified sampling interval, they approached the selected child’s caregiver about the survey to obtain parental permission. If permission was obtained, they circled the number in the column and proceeded with enrollment (see figure, second row). If the selected child could not be enrolled due to refusal or no caregiver present to obtain permission, the staff marked this in the column and indicated the reason (see figure, first row not enrolled due to refusal). At this point the survey staff attempted to enroll the next available child (e.g., 11^th^ child in below example) then proceeded as before with the counting and sampling per the specified sampling interval.


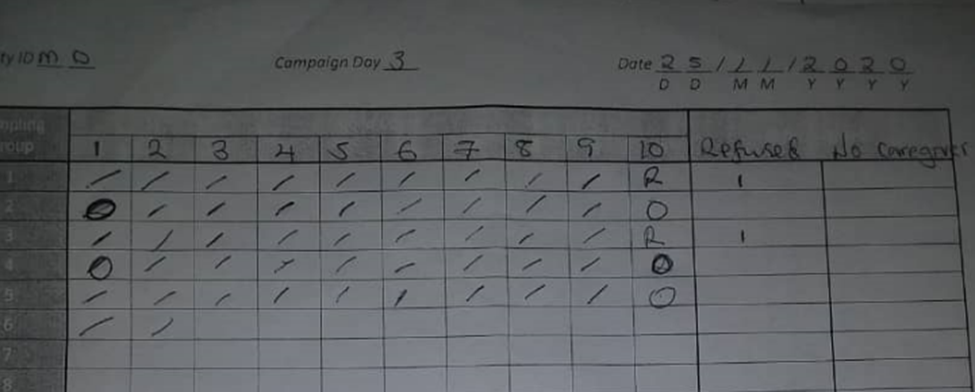


Only those children whose caregivers were interested at the sampling stage and proceeded to the survey table were assigned a study ID and entered in the study tablet. This reduced documentation however meant that the sampling log was key for evaluating participation rates (enrolled out of approach) and documenting reasons for non-enrollment at the sampling stage.

Implementation

Each survey team decided how to distribute responsibilities between staff members and how to engage with the vaccinators and other health facility staff at the site. Responsibilities included counting and sampling children, approaching the caregiver and introducing the survey, obtaining parental permission, administering the questionnaire using a tablet-based form (REDCap) and collecting a dried blood spot sample by fingerprick.

Daily Tracking Log

At the end of each day survey staff completed a Tracking Log to compile daily information about the campaign and serosurvey. Details about the campaign included how long the site was open for vaccination, how many vaccination teams were present, total number of children vaccinated, an assessment on volume of children (fewer than expected, as expected, more than expected), and any disruptions (e.g., rumors, weather issues). For staff working at outreach locations that varied throughout the week they also recorded information on where they were located that day (e.g., church, market).  Using the Sampling Log they summarized the number of children approached for the survey, number enrolled, and number not enrolled due to refusal or no caregiver and recorded this information in the Tracking Log. Photos of the logs were uploaded to a survey WhatsApp group then the central team compiled all information using an electronic form.


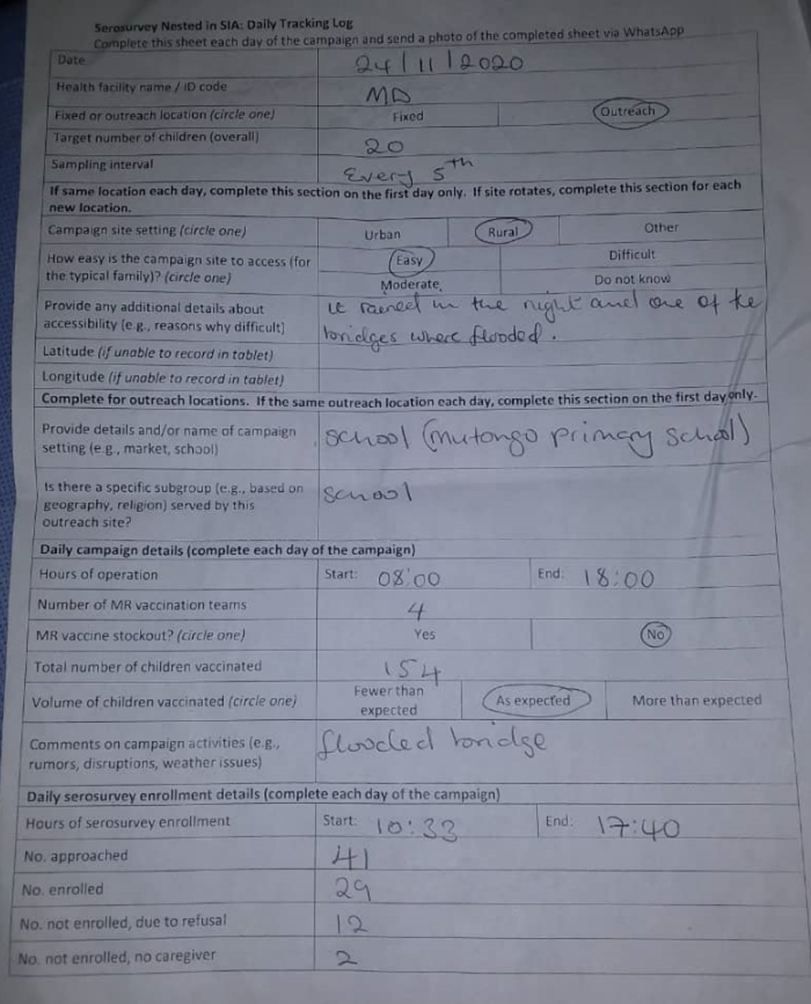


Sampling interval

How the survey staff adjusted the sampling interval varied by survey location. Some survey teams adjusted the interval during the day as the volume of children changed, while others used the pre-specified interval until a change was communicated. For facilities with rotating outreach locations, particularly in Choma District, vaccinators worked together to quickly vaccinate as many children as possible upon arrival at a location. In these situations, it was logistically difficult for the survey team to maintain the specified sampling interval so they adjusted as needed based on staffing capacity. On the third day of the SIA we learned that some SIA locations in Choma District planned to continue vaccinating on the sixth day. We adjusted the sampling intervals and daily maximum at those facilities to slow enrollments on the subsequent days to capture children vaccinated on the sixth day. While we assumed the same number of children vaccinated per day for planning purposes, we observed the volume of children was lower during the initial days of the SIA.

There may have been issues with projections or assumptions on which of the facility’s SIA locations the community would be more likely to attend. There may have also been issues in how the microplans were interpreted in the survey planning process, as the targets seemed closer to the observed estimates in Choma compared to Ndola.

Daily Data Summaries

A summary report was developed in advance of data collection to prepare daily reports to monitor data enrollment by site and provide near real-time feedback on vaccination status of children attending the SIA to our collaborators at the Ministry of Health.


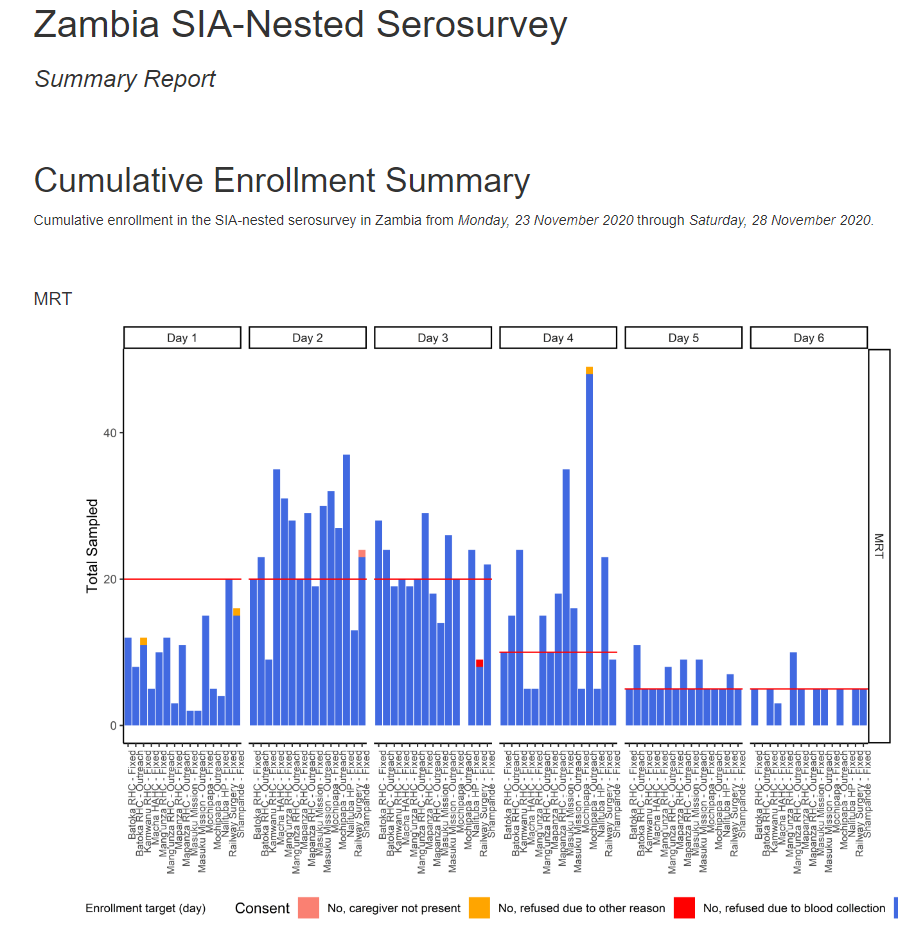


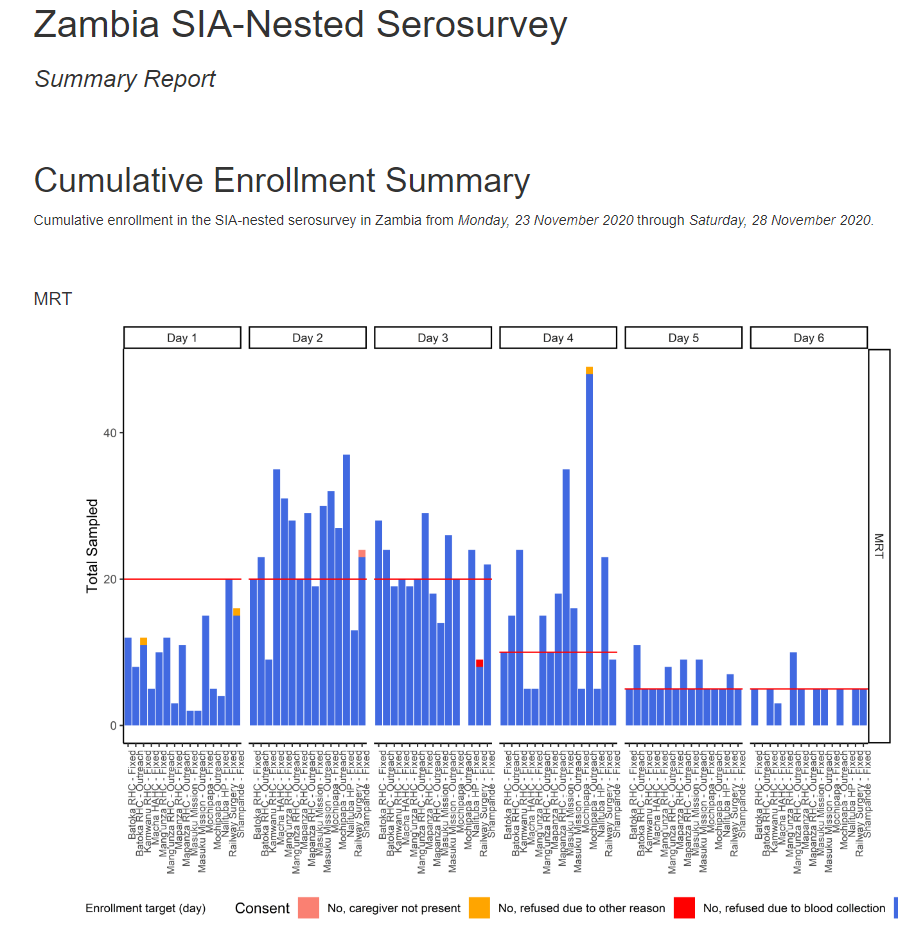


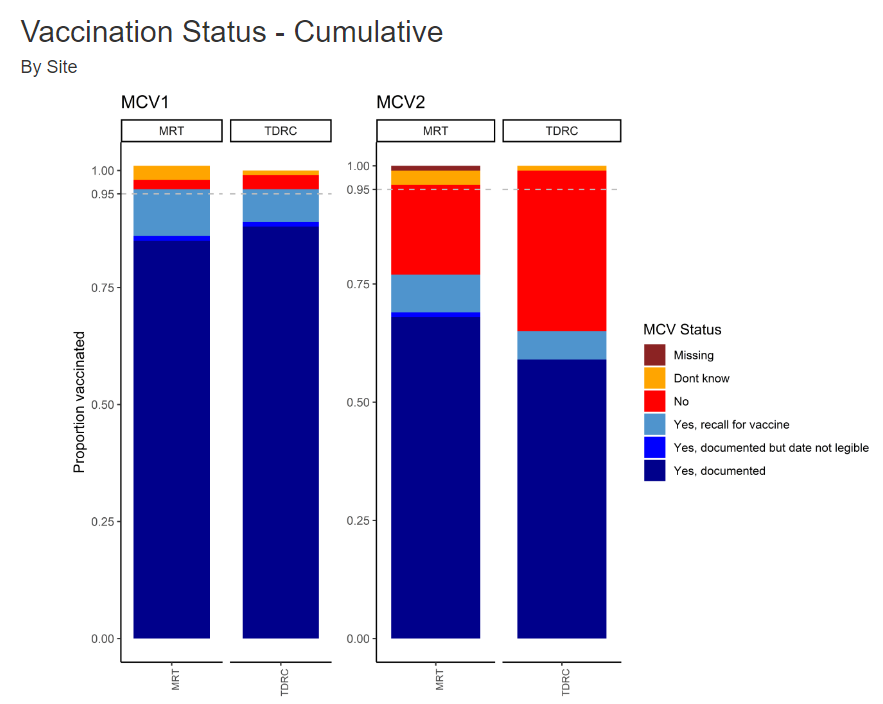

Supplement: S1 Appendix — (DOCX) [file pgph.0002985.s004.docx]
